# Total Phenol Content and Antioxidant Activity of Different Celta Pig Carcass Locations as Affected by the Finishing Diet (Chestnuts or Commercial Feed)

**DOI:** 10.3390/antiox10010005

**Published:** 2020-12-23

**Authors:** Noemí Echegaray, Paulo E. S. Munekata, Juan A. Centeno, Rubén Domínguez, Mirian Pateiro, Javier Carballo, José M. Lorenzo

**Affiliations:** 1 Centro Tecnológico de la Carne de Galicia, Rúa Galicia Nº 4, Parque Tecnológico de Galicia, San Cibrao das Viñas, 32900 Ourense, Spain; noemiechegaray@ceteca.net (N.E.); paulosichetti@ceteca.net (P.E.S.M.); rubendominguez@ceteca.net (R.D.); mirianpateiro@ceteca.net (M.P.); 2 Área de Tecnología de los Alimentos, Facultad de Ciencias de Ourense, Universidad de Vigo, 32004 Ourense, Spain; jcenteno@uvigo.es (J.A.C.); carbatec@uvigo.es (J.C.)

**Keywords:** Celta pig breed, animal diet, chestnut, commercial feed, muscle tissue, liver, total phenol content (TPC), total flavonoids, antioxidant activity

## Abstract

The objective of this research was to evaluate the total phenol content, total flavonoids, and antioxidant activity of chestnuts (*Castanea sativa* Mill.) and commercial feed employed in the finishing diet of the Celta pig breed and analyze the effect of the feeding (chestnuts vs. commercial feed) in the finishing diet on total phenol content and antioxidant activity of *Longissimus thoracis et lumborum*, *Psoas major*, and *Biceps femoris* muscles and liver of the Celta pig breed. The antioxidant activity of the feed and animal tissue was investigated using three antioxidant methods (2,2-Diphenyl-1-picrylhydrazyl (DPPH) radical scavenging activity, 2-2′-Azino-di-[3-ethylbenzthiazoline sulfonate] (ABTS) radical scavenging activity, and ferric reducing antioxidant power (FRAP) assay). The determination of the total phenol content and total flavonoids showed that chestnut had a significantly lower concentration than commercial feed in these compounds (130.00 vs. 312.89 mg gallic acid equivalents/100 g fresh weight and 8.58 vs. 32.18 mg catechin equivalents/100 g fresh weight, respectively). However, the results displayed that chestnuts had a higher antioxidant activity when compared with the commercial feed through the DPPH and ABTS methods (1152.42 vs. 957.33 µg Trolox equivalents/g fresh weight, and 9379.74 vs. 7613.44 µg Trolox equivalents/g fresh weight, for DPPH and ABTS assay, respectively), while the antioxidant activity measured by the FRAP assay turned out to show higher values for commercial feed (1777.49 and 1946.09 µmol Fe^2+^/100 fresh weight for chestnut and commercial feed, respectively), although significant differences were only found in the ABTS assay. On the other hand, the present study found that chestnut significantly reduces the total phenol content and declines the antioxidant activity of *Longissimus thoracis* et *lumborum*, *Psoas major*, and *Biceps femoris* muscles and liver of the Celta pig breed. Finally, it has been found that liver is the location that has the best antioxidant characteristics compared to any muscle, regardless of diet utilized.

## 1. Introduction

During the shelf life and storage of food, oxidative processes generate the degradation of pigments, lipids, and proteins that simultaneously can contribute to the deterioration of color, texture, taste, and nutritional value of the nourishments [1,2,3]. This is the case for fresh meat, which, due to its chemical characteristics, is prone to oxidative deterioration [4,5,6]. For their part, antioxidants are chemical compounds capable of donating hydrogen to the free radicals, which can prolong the shelf life of meat, delaying the oxidation of lipids, loss of color, and microbial growth [7]. Therefore, with the purpose of minimizing the decline process in meat and meat products, the use of additives with antioxidant properties has been widely used in meat processing [8,9,10,11]. Specifically, the current trend in the meat industry is the use of natural antioxidants to the detriment of synthetics compounds on account of their toxic potential [12]. In this regard, vegetal resources are a rich origin of polyphenolic compounds, which are secondary metabolites of plants with antioxidant properties [13,14]. This is the case of the chestnut (*Castanea sativa.* Miller) fruit, because this kernel is an important source of phenol compounds such as phenolic acids (chlorogenic, coumaric, ellagic, ferulic, and gallic acid), flavonoids (apigenin, rutin, and quercetin), and tannins [15,16,17,18].

On the other hand, the population is currently also highly interested in the consumption of meat and meat products obtained from ancient autochthonous genetic types. These occurrences respond to the fact that the breeding and management of autochthonous breeds is conditioned to animal welfare, considering that the livestock raised in this way have the ability to express the natural behavior of the species, at the same time that the high quality and qualitative and organoleptic characteristics of meat thrive [19,20]. On this matter, the Celta pig breed originally from Galicia (northwest Spain) takes on special interest, since these pigs have traditionally been reared in extensive or semi-extensive systems employing natural food sources such as chestnut. In this sense, the products obtained from pigs fed chestnuts are characterized by their high quality in terms of the intense fat infiltration into lean, their healthier fat, and their succulent meat [21,22]. More concretely, the use of chestnuts has been shown to increase the degree of unsaturation of fat in meat and meat products [21,23,24,25]. This characteristic, although it is beneficial for health, could have a detrimental effect on meat, because unsaturated fatty acids are easily susceptible to oxidation [3]. Nevertheless, this situation has not been displayed in preliminary studies [24,26,27,28]. In this way, Cobos et al. [27] observed that the inclusion of 15% chestnut flour in a pig diet decreased the lipid oxidation of a dry-cured pork foreleg. Similarly, Pugliese et al. [24] demonstrated that the use of chestnuts during the last three months of pig feeding prevented the risk of lipid oxidation, since they found low values of malonaldehyde (MDA) in meat from pigs fed with this fruit. Furthermore, a previous trial of our research group conducted on cooked meat found that the inclusion of chestnut in the finishing diet of the Celta pig exerted a protective effect on lipids of the *Longissimus thoracis* et *lumborum* muscle [28]. Additionally, other studies showed that the use of chestnuts in pig fattening did not modify lipid oxidation in both fresh meat and its derivatives [29,30,31].

These effects could be due to the phenolic compounds of the chestnut fruit, which once ingested by the pig could be accumulated in the animal tissues and exert a protective effect on the meat lipids, ameliorating the shelf life and the technological quality of meat [20,32,33]. Notwithstanding, despite these findings, the information on the relationship between the antioxidant characteristics of the diet containing chestnuts and the total phenol content as well as the antioxidant capacity of pig tissues is nonexistent. Therefore, the purpose of the present study was to analyze the total phenol content, total flavonoids, and antioxidant activity of chestnuts (*Castanea sativa* Mill.) and commercial feed used in the finishing diet of Celta pig breed, as well as to investigate the effect of the feeding (chestnuts or commercial feed) in the finishing diet on total phenol content and antioxidant activity in diverse locations in the carcass (*Longissimus thoracis* et *lumborum*, *Psoas major*, and *Biceps femoris* muscles, and liver).

## 2. Materials and Methods

### 2.1. Experiment Design and Pig Slaughter

A total of 18 Celta pigs (10 males and 8 females) raised in a semi-extensive system were employed for this work. The piglets were weaned until 40 days and were vaccinated and deparasitized according to the standard protocols. After suckling, the pigs were reared in a semi-extensive regime with a livestock density of 12 animals per hectare and fattened with a commercial compound. Males and females were castrated under anesthesia and additional prolonged analgesia at the age of 2 and 3 months, respectively, in accordance with the Council Directive 2008/120/EC [34]. The pigs were randomly split into 2 distinct groups of 9 animals (5 males and 4 females) at the age of 8 months for their special feeding during the finishing period (4 months). The animals were maintained in distinct portions of land, guaranteeing that there was not any other vegetation which pigs had access to. One of the groups, after eating a transition mixed diet composed of commercial compound feed and chestnuts (1.5 kg and 3 kg per animal and day, respectively) during the ninth month, were fed with 6 kg of chestnuts per animal and day in the remaining three months previously to slaughter. The pigs of the other group were fattened with 3 kg of commercial feed per animal and day during the 4 months prior to the slaughter age.

At the end of the fattening period, livestock was transported 80 km to a commercial abattoir (Frigolouro, Porriño, Pontevedra, Spain) and were kept for 12 h with free access to water but not to nourishment. Animals were slaughtered with a mean live weight of 107.53 ± 8.26 kg for pigs fed with chestnut and 115.41 ± 9.15 kg for pigs fed with commercial feed (*P* > 0.05). The slaughtering method was carried out by electrical stunning and exsanguination. Subsequently, pigs were scalded, skinned, and eviscerated following standard commercial procedures. Straight away, carcasses were chilled at 4 ± 1 °C in a cold chamber for 24 h. After the cooling period, samples from *Longissimus thoracis et lumborum*, *Psoas major*, and *Biceps femoris* muscles and from the liver from each carcass were extracted and preserved chopped in vacuum-sealed bags at −80 ± 2 °C until processing.

### 2.2. Finishing Diet Material

Chestnut samples were obtained from a mix of the Spanish cultivars Amarelante, Famosa, Longal, and Judía. The fruits were harvested between the months of October and November 2015 from orchards of different points in the provinces of Lugo, Ourense, and León (northwest Spain). After collection of samples, chestnuts were stored in a refrigerator at 4 ± 1 °C for a maximum of 3 days until they were hand-peeled, separating the tegument tissue. Continuously, the peeled fruit was chopped and stored in vacuum-sealed bags at −80 ± 2 °C until subsequent extraction.

Commercial pig feed composed of barley, roasted and decorticated soybean-extracted flour, maize, wheat, bran, winemaking lees and maize soluble, beet molasses, calcium carbonate, phosphate, mineral dicalcium, soybean hulls, vegetable palm oil, and sodium chloride and supplemented with trace minerals, vitamins, provitamins, and chemically defined substances, which have an effect analogous to Vitamins A, D_3_, and E, were supplied by Sarriana de Piensos S.A. (Lugo, Spain). Composition of the commercial feed is shown in Table 1. Upon receipt, this commercial nourishment was chopped and stored in vacuum-sealed bags at −80 ± 2 °C until processing.

The chemical, cholesterol, retinol, and fatty acid composition and the energy of the two different diets used in the finishing diet of Celta pigs (chestnuts and commercial feed) were reported in previous studies [23,30].

### 2.3. Extract Preparations

The extracts utilized to determine the phenolic compounds, flavonoids content (determined only in pig feed and not in meat samples), as well as the antioxidant assays were carried out following the method proposed by Santos et al. [35], with slight modifications. Succinctly, three grams of previously chopped and thawed sample were disrupted for 60 s using an IKA T25 digital Ultra-Turrax (IKA^®^-Werke GmbH & Co. KG, Staufen, Germany) in 20 mL of CH_3_OH: H_2_O (80:20, *v*/*v*). Then, homogenate was stirred at 50 rpm on a rocking shaker (SW-3D-E, OVAN, Barcelona, Spain) for 15 min, at room temperature. After stirring, samples were placed in an ultrasound water bath apparatus (Bransonic^®^ 8510E-DTH, Danbury, CT, USA) for 15 min at 25 °C. Subsequently, they were centrifuged at 14,000× *g* and 4 °C during 15 min, and supernatant was separated and filtered through 0.45 µm pore filter (Acrodisc^®^ LC PVDF syringe filter; Pall Gelman Laboratory, Montreal, QC, Canada). The methanolic extracts were stored protected from direct exposition to light at −80 °C prior to their corresponding analysis.

### 2.4. Determination of Total Phenol Content (TPC)

Total phenol content was estimated following the Folin–Ciocalteu method for total phenolics, which is substantiated on the colorimetric oxidation/reduction reaction of phenols. This assay was carried out according to the method initially described by Singleton and Rossi [36], with some modifications. Basically, 500 μL of methanolic sample extracts (diluted for convenience) were added to 2.5 mL of Folin–Ciocalteu: H_2_O reagent (1:10, *v*/*v*) and mixed before adding 2.0 mL of 7.5% (*w*/*v*) NaCO_3_. The mix was incubated in a water bath at 45 °C and absorbance at 765 nm was read employing a spectrophotometer UV-1800 (Shimatzu Corporation, Kyoto, Japan) after cooling at room temperature during 30 min. Readings were compared with a standard curve of gallic acid (ranged from 0 to 100 mg gallic acid/L), being the total phenol content expressed as milligrams of gallic acid equivalents (GAE) per 100 g of fresh weight (FW) of pig feed or pig tissue.

### 2.5. Determination of Total Flavonoids

Total flavonoids were determined in chestnut and commercial feed by the aluminum chloride colorimetric method initially described by Zhishen et al. [37], with slight changes proposed by Rodrigues et al. [38]. Firstly, 1 mL of the methanolic extract (diluted for convenience) was mixed with 4 mL of distilled water and 300 µL of a 5% (*w*/*v*) NaNO_2_ solution. After 5 min, 300 µL of a 10% (*w*/*v*) AlCl_3_ solution was mixed in, and 1 min later, 2 mL of 1 M NaOH and 2.4 mL of distilled water were also added. The solution was mixed in a vortex and the absorbance was read at 510 nm using a spectrophotometer UV-1800 (Shimatzu Corporation, Kyoto, Japan). (+)-Catechin (ranged from 0.250–2.500 mM) was employed to calculate the standard curve and the results were expressed as mg of (+)-catechin equivalents (CAE) per 100 g of fresh weight (FW) of pig feed or pig tissue.

### 2.6. 2,2-Diphenyl-1-Picrylhydrazyl (DPPH) Radical Scavenging Activity

DPPH radical scavenging activity is a technique considered a standard for the in vitro evaluation of antioxidants, which is widely employed for the evaluation of free radical scavenging potentials of distinct compounds [39]. This method was accomplished based on the procedure described by Brand–Williams et al. [40], with some modifications. Briefly, 100 µL of the methanolic sample extract (diluted for convenience) were incubated at 37 °C for 10 min after adding 3900 µL of a methanolic DPPH solution (60 µM) prepared daily and mixed in a vortex. Absorbance was read at 515 nm in a spectrophotometer UV-1800 (Shimatzu Corporation, Kyoto, Japan). The DPPH radical scavenging activity of the samples was calculated from a standard curve of Trolox (ranged from 0 to 1.2 mM Trolox) and expressed as µg Trolox equivalents (TE)/g fresh weight (FW) of pig feed or pig tissue.

### 2.7. 2-2′-Azino-di-[3-Ethylbenzthiazoline Sulfonate] (ABTS) Radical Scavenging Activity

ABTS discoloration assay of samples was determined following the adapted Trolox-equivalent antioxidant capacity (TEAC) method firstly explained by Re et al. [41], with slight changes. This method is substantiated on the ability of antioxidants to quench the long-lived ABTS radical cation, a bluish-green chromophore with a specific absorption line at 734 nm. The ABTS radical cation was produced by reacting 7.00 mM ABTS stock solution with 2.45 mM potassium persulfate, a strong antioxidant agent. The mixture was incubated in the dark at room temperature for 12–16 h before use. Previously to being utilized, the ABTS solution was diluted with distilled water to reach an absorbance of 0.700 ± 0.020 at 734 nm, and equilibrated at 30 °C. Following, an aliquot of 20 µL of methanolic extracts of the samples (diluted for convenience) was added to 980 µL of the working ABTS solution and the absorbance was read at the specific wavelength (734 nm) utilizing a spectrophotometer UV-1800 (Shimatzu Corporation, Kyoto, Japan) after 10 min in darkness. A standard curve of Trolox (ranged 0–2.0 mM Trolox) was employed for the quantification of the ABTS radical scavenging activity and outcomes were expressed as µg Trolox equivalents (TE)/g fresh weight (FW) of pig feed or pig tissue.

### 2.8. Ferric Reducing Antioxidant Power (FRAP) Assay

The FRAP assay was carried out following the method previously described by Benzie and Strain [42], with brief changes. This method is based on the ability of certain antioxidants species to reduce iron (III) to the ferrous form (II) in acid medium, which develops a blue color. In this way, this test measures the development of an intense navy-blue color that corresponds to the formation of Fe^2+^-2,4,6-Tri(2-pyridyl)-1,3,5-triazine (TPTZ) complex from the colorless oxidized Fe^3+^-TPTZ complex, which has a maximum absorbance at 593 nm. FRAP solution was freshly prepared by dissolving an acid solution of 10 mM 2,4,6-tripyridyl-s-triazine (TPTZ) in 40 mM HCl, an aqueous solution of 20 mM FeCl_3_: 6H_2_O, and a buffer solution of 300 mM acetate buffer (pH 3.6) at a ratio of 1:1:10 (v:v:v). Afterwards, to 900 µL of FRAP reagent 90 µL of distilled water and 30 µL of the methanolic extracts (diluted for convenience) were added. This mixture was incubated for 20 min at 37 °C in darkness and the absorbance was measured at 593 nm employing a spectrophotometer UV-1800 (Shimatzu Corporation, Kyoto, Japan) after cooling at room temperature for 15 min. Ferrous sulphate solutions (FeSO_4_·7H_2_O), ranging from 0–2 mmol, were utilized to obtain the calibration curve. The FRAP values were expressed as µmol Fe^2+^/100 g fresh weight of pig feed or pig tissue.

### 2.9. Statistical Analysis

In each feed and in each location of each pig carcass, determinations were made in triplicate for each parameter. With the purpose of analysis, the differences between chestnut and commercial feed, as well as the sway of feeding and muscle location in Celta pigs on the different parameters studied, an analysis of variance (ANOVA) using the General Linear Model (GLM) procedure of the SPSS package version 23.0 (IBM SPSS, Chicago, IL, USA) was accomplished. Following, Duncan’s test was carried out. Correlations between variables were determined employing the Pearson’s linear coefficient implemented with SPSS package, version 23.0. *P* values were determined and levels of significance were expressed as *P* < 0.05, *P* < 0.001 or *P* < 0.001.

## 3. Results and Discussion

### 3.1. Total Phenol Content of Feed Used in the Finishing Diet of Celta Pigs

**Figure 1:**
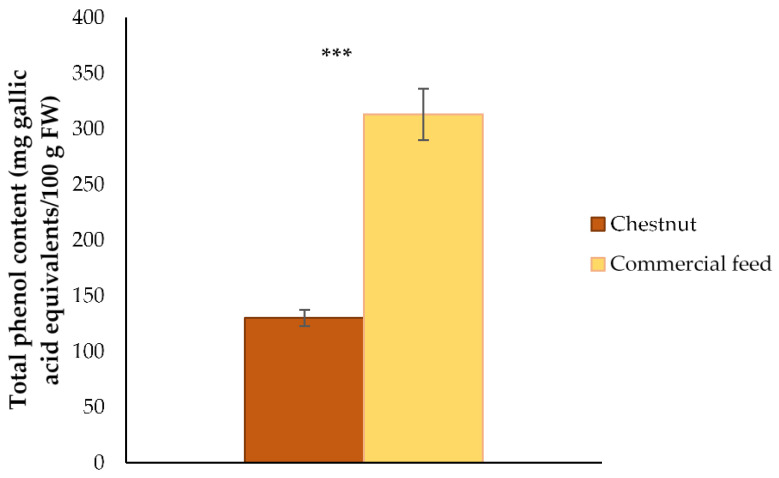
Total phenol content of chestnut and commercial feed used in the finishing diet of Celta pigs (mean ± standard error of three determinations in each feed). *** (*P* < 0.001). FW: fresh weight.

Total phenol content (TPC) are mainly composed of phenolic acids, flavonoids, and anthocyanins. In the case of chestnut fruit, some of the most important phenolic substances found are gallic, chlorogenic, coumaric, ferulic and ellagic acids, and catechin, quercetin, and rutin [18], while in commercial feed it depends on the nature of grains and supplements used in its preparation. Figure 1 displays the results corresponding to the TPC of chestnut and commercial feed employed in the finishing diet of Celta pig as mg GAE/100 g FW.

The values obtained for the TPC of chestnut (130.00 mg GAE/100 g FW) were similar to those displayed by Hernández Suárez et al. [15], who found mean values of 124 mg GAE/100 g FW for 21 different varieties of chestnut from Tenerife. However, other authors obtained results that were further from ours. For example, Kalogeropoulos et al. [43] and Nazzaro et al. [18] showed lower amounts of TPC for chestnuts from distinct regions. Concretely, Kalogeropoulos et al. [43] observed a TPC of 43.0 mg GAE/100 g FW for chestnuts from a cultivar from Crete Island. For their part, Nazzaro et al. [18] determined a value of 76.3 mg GAE/100 g FW for “Palomina” cultivar from Italy. In the case of a study conducted by Neri et al. [44], even lower values were observed for three different commercial chestnuts ecotypes from Italy, ranging from 5.1 to 7.9 mg GAE/100 g FW. On the contrary, other works about chestnuts’ fruit report a TPC higher than that determined in the present research. This is the case of a work carried out by Özel [45], where values between 137.8–386.4 mg GAE/100 g FW were found in chestnuts belonging to 4 different origins of Turkey. Moreover, Carocho et al. [46] found even higher amounts in chestnuts harvested in the Portugal region, values ranging among 361 and 816 mg GAE/100 g chestnut. Although, it should be noted that samples were previously lyophilized, and the highest amounts of total phenols were shown in chestnuts that were treated prior with different types of radiation. Additionally, Otles and Selek [47] found a TPC between 690–2140 mg GAE/100 g DW in raw chestnuts from three distinct varieties from Turkey. This content is quite high with respect to those found in our study, but it highlights that these authors have expressed the TPC in dry weight, therefore, this difference could be too high. The high variability in TPC reported in literature for chestnuts seems to be a consequence of differences in varieties and geographical origins (edaphic-climatic conditions of cultivation). Additionally, the differences in the procedures used for TP extraction, which involved different solvents, extraction times, temperatures, and auxiliary treatments (e.g., ultrasounds) in some cases, or previous treatments such as irradiation or lyophilisation, could also be partially responsible for these variabilities.

With respect to commercial pig feed, this research has shown that this diet possessed a TPC of 312.89 mg GAE/100 g FW. These values are higher than those displayed in other studies, where values between 110–116 mg GAE/100 g DW were found for commercial concentrates intended for pig feeding [48,49]. Nevertheless, the higher presence of total polyphenols in commercial feed employed in this research could be due to the existence of certain cereals such as barley and maize. In this way, barley has recently been considered as an ingredient for the production of functional foods [50]. This is so since this cereal possesses high amounts of bioactive substances such as tocopherols, tocotrienols, β-glucans, and different kinds of phenolic compounds like cinnamic and benzoic acid derivatives, proanthocyanidins, quinones, flavonols, chalcones, flavones, flavanones, and amino phenolic compounds [51,52]. More concretely, barley cereal owns higher amounts of phenolic compounds (0.2–0.4%) than other cereal grains [53]. For instance, Suriano et al. [54] showed TPC ranging from 192.9 to 291.7 mg GAE/100 g in whole-grain barley grown in Southern Italy, which are close to the values obtained in this study for the commercial feed (312.89 mg GAE/100 g FW). For their part, Han et al. [55] displayed a mean value of TPC of 203.314 mg GAE/100 g for 223 distinct barley genotypes cultivated in southeast China. Additionally, the commercial feed employed in the finishing diet of Celta pig also contained maize among the three main ingredients. This cereal has been found to exhibit a TPC higher than wheat, oat, and rice [51]. Specifically, Lopez-Martinez et al. [56] found a TPC content for four types of corn (white, red, blue, and purple pigmented) ranging from 170.1 to 1760 mg GAE/100 g of cereal, values among which are the TPC of the commercial feed analyzed in this work. Within these ranges is also the TPC found by Van Hung [51], who evidenced quantities of 264.54 mg GAE/100 g of maize for this parameter. Finally, Saikaew et al. [57] displayed a TPC of 295.98 mg GAE/100 g DW in untreated maize, which was similar to those found in the commercial pig feed supplied in this study.

In addition, even though chestnuts are an important source of phenol compounds [15], this study has determined that commercial feed provides a significantly (*P* < 0.001) higher TPC to the pig diet when comparing this nourishment with chestnuts (312.89 vs. 130.12 mg GAE/100 g FW). This finding disagrees with those described previously by Tejerina et al. [48] and González and Tejeda [49] since these authors observed that the concentrated feed used in the pig diet had a lower content of polyphenols than natural nourishments (namely, grass and acorns) as could be expected in the case of chestnut.

### 3.2. Total Flavonoids of Feed Used in the Finishing Diet of Celta Pigs

**Figure 2:**
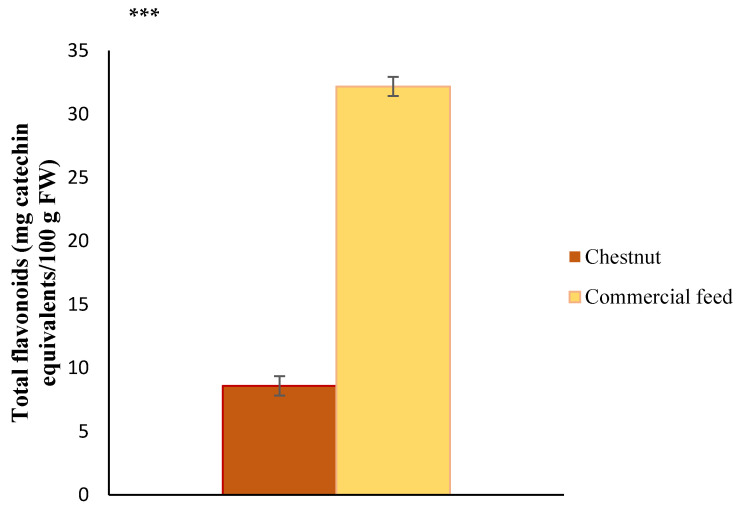
Total flavonoids of chestnut and commercial feed used in the finishing diet of Celta pigs (mean ± standard error of three determinations in each feed). *** (*P* < 0.001). FW: fresh weight.

Values of total flavonoids of chestnut and commercial feed used in the finishing diet of Celta pigs are displayed in Figure 2 as mg CAE/100 g FW. As we can see, the total flavonoids found in chestnuts’ fruit in this research were 8.58 mg CAE/100 g FW. These outcomes stand out for their low value when compared with those obtained by other authors. For example, Carocho et al. [46] found amounts of total flavonoids between 24 and 234 mg CAE/100 g lyophilized chestnuts from Portugal. For their part, Antonio et al. [58] displayed values that exceeded 100 mg CAE/100 g DW also in Portuguese chestnuts, which increased after storage to amounts exceeding 700 mg CAE/100 g DW. Our values also contrast with those displayed by Živković et al. [59], who determined a flavonoid content of 170 mg CAE/100 g DW in chestnuts from Bosnia and Herzegovina. Additionally, Dinis et al. [60] found higher values of these compounds, demonstrating that different ecotypes of this kernel grown in Portugal had a total amount of flavonoids between 480 and 6720 mg CAE/100g DW. As occurred for the TPC, these discordant values could be due to the differences between the distinct varieties, origins, and edaphic-climatic conditions of cultivation of chestnuts compared [45,47,60] and also to the different procedures used for extraction or preliminary treatment.

Regarding commercial feed, this work showed that this diet has total flavonoids of 32.18 mg CAE/100 g FW. The outcomes obtained in this research cannot be compared with other studies, since no bibliography has been found on the matter for commercial compound pig feed. Despite this lack of data, the outcomes obtained for total flavonoids of commercial pig feed have been compared with the presence of these biocompounds in barley and maize, since they are two of the three main feed ingredients. In this way, similar amounts of total flavonoids were obtained for maize (the third ingredient of the feed) by Saikaew et al. [57]. Specifically, they found total flavonoid values between 34.21 and 36.03 mg CAE/100 g DW for fresh corn. On the other hand, in the case of barley, the values shown for the total flavonoids by Han et al. [55] turned out to be higher than those obtained in the case of the commercial feed supplied in this study (80.04 vs. 32.18 mg CAE/100 g).

Additionally, it has been observed that the commercial feed showed significantly (*P* < 0.001) higher amounts of total flavonoids than chestnuts analyzed in the present work (32.18 vs. 8.58 mg CAE/100 g FW). Notwithstanding, the values obtained for this swine diet are far from the values showed by other authors for chestnuts, ranging from 100 to 6720 mg CAE/100g DW [58,59,60]. Although, it is true that said values would be within the range of total flavonoids obtained by Carocho et al. [46] for lyophilized chestnuts (24 and 234 mg CAE/100 g).

### 3.3. Total Phenol Content of Different Celta Pig Locations

**Figure 3:**
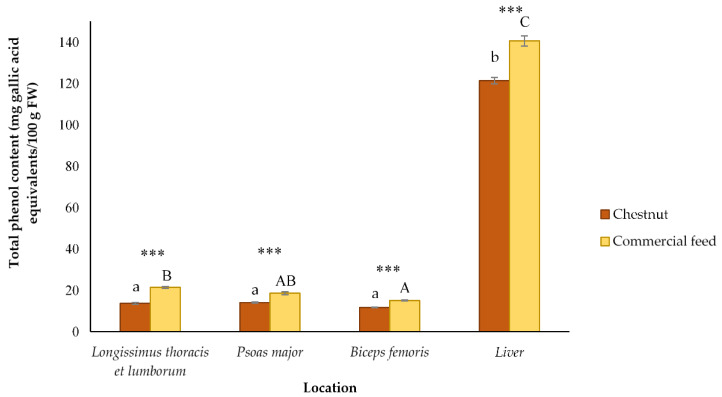
Effect of the inclusion of chestnut in the finishing diet and of location in the carcass on total phenol content of Celta pig tissues (mean ± standard error of nine carcasses in each feeding group). *** Significant differences (*P* < 0.001) influenced by feeding in each carcass location. ^a^^,^^b^ Means within the same stick for chestnut fed pigs not followed by a common small letter differ significantly (*P* < 0.05) (differences associated to the location). ^A–C^ Means within the same stick for commercial feed fed pigs not followed by a common uppercase letter differ significantly (*P* < 0.05) (differences associated to the location). FW: fresh weight.

The presence of compounds with antioxidant properties such as phenols in meat improves the nutraceutical value and the technological quality of meat [20]. Values corresponding to the TPC of the four different locations are presented in Figure 3, expressed as mg GAE/100 g FW. The outcomes obtained for muscle tissue (ranging from 11.70 to 14.06 mg GAE/100 g FW for chestnut pigs and from 15.08 to 21.43 mg GAE/100 g FW for commercial feed pigs) were similar to those found by other authors, who showed levels ranging between 12.64 and 24.18 GAE/100 g FW for Iberian pig muscles [48,61]. On the contrary, Simonetti et al. [20] obtained a TPC for the *Longissimus lumborum* muscle of Italian pigs much higher (between 133.62 and 122.39 mg GAE/100 g FW) than that revealed for the muscles of the present study. However, the values obtained by these authors were very similar to those shown by the liver in both diets in the present work (121.81 mg GAE/100 g FW for liver of pigs fed with chestnut and 140.55 mg GAE/100 g FW for livers of pigs fed with commercial feed).

As can be seen, the inclusion of chestnut fruit in the finishing diet significantly (*P* < 0.001) reduces the values of TPC for all the locations analyzed. This reduction could be related to the lower values of TPC and total flavonoids detected in chestnut compared to the amounts found in commercial feed (Figure 1 and Figure 2), since the presence of phenolic compounds in animal tissues is mainly relegated to the deposition of these substances after the ingestion of plant resources which have said bioactive compounds in their composition [33]. Thereby, the higher content of particular antioxidants in commercial feed could be associated with the greater deposition of these antioxidants in the muscle and liver from pigs fed with this elaborated nourishment, and consequently with a higher TPC (Figure 3). This possible relationship between the amount of phenolic compounds ingested and the phenolic compounds deposited in pig tissues was previously suggested by other authors [61,62,63,64]. On this matter, Tejerina et al. [61] observed that a higher TPC in acorn and grass used in the feeding of Iberian pigs provided a significantly higher concentration of these compounds in the *Longissimus dorsi* and *Serratus ventralis* muscles. In the same line, González et al. [63] proposed a similar trend in Iberian pigs fed under three different regimes. Concretely, they observed that the highest TPC in the adipose subcutaneous tissue corresponded to pigs raised on acorn and grass, precisely the feeds that had the highest TPC of all the diets tested. Furthermore, Tejerina et al. [48] also found that pigs fattened on a diet richer in polyphenolic compounds (grass and acorn) showed a higher TPC in muscle than pigs fed a concentrate compound that was poorer in these substances. Additionally, Flis et al. [62] proposed that the TPC of pork tissues could be improved by increasing the phenolic content in the supplied diet. Contrary to all these findings, González and Tejeda [49] found that the different content of total phenols in distinct dietary treatments (ranging from 110 to 1694 mg caffeic acid/100 g) did not significantly alter the deposition of phenolic substances in the *Longissimus dorsi* muscle of Iberian pigs (TPC levels ranging from 16.22 to 19.58 mg caffeic acid/100 g).

However, the relationship between the antioxidant compounds present in the diet and their deposition in the animal’s muscle has also been reported in other species such as poultry and rabbits. In this way, Jang et al. [65] observed that supplementation with a dietary medicinal herb extract (rich in polyphenols) at different concentrations (0.3 and 1.0%) increased the total polyphenol content of Broiler chicken breasts when compared to a control diet (9.56 and 9.92 vs. 4.88 mg GAE/100 g FW). Furthermore, Perna et al. [64] found that a rabbit diet supplemented with cauliflower leaf powder resulted in a significant increase in TPC in *Longissimus lumborum* muscle compared to a standard diet (5.98 vs. 4.85 mg GAE/100 g).

On the other hand, the effect of pig location affects TPC in a similar and significant (*P* < 0.001) way in both diets. Thus, lowest TPC was found for *Biceps femoris* muscle in both diets (11.70 mg GAE/100 g FW for pigs fed with chestnuts, and 15.08 mg GAE/100 g FW for pig fed with commercial feed). Meanwhile, *Longissimus thoracis* et *lumborum* and *Psoas major* muscles showed intermediate values for TPC (13.68 and 14.06 mg GAE/100 g FW in the pigs fed with chestnuts and 21.43 and 18.60 mg GAE/100 g FW in the pigs fed with commercial feed, respectively). On the contrary, the highest TPC was found for the liver (121.38 and 140.55 mg GAE/100 g FW for chestnut and commercial feed pigs, respectively). This greater presence of phenolic compounds in liver compared to the muscle tissue could be similar to what occurs with other nutrients, since, for instance, the liver contains important amounts of some vitamins such as retinol (Vitamin A), riboflavin (Vitamin B_2_), niacin (Vitamin B_3_), pyridoxine (Vitamin B_6_), folacin (Vitamin B_9_), cobalamin (Vitamin B_12_), ascorbic acid (Vitamin C), and tocopherol (Vitamin E) [66,67,68,69,70].

### 3.4. Antioxidant Activity of Feed Used in the Finishing Diet of Celta Pigs

Various biochemical trials were employed to screen the antioxidant properties: scavenging activity on DPPH and ABTS radicals (considering the decrease in DPPH and ABTS radical absorption after exhibition to radical scavengers) and FRAP assay (the antioxidants present in the samples reduce the ferric ion to its ferrous form of the Fe(III)/tripyridyltriazine complex). Table 2 displays the DDPH and ABTS radical scavenging activity, as µg TE/g FW, and FRAP values, as µmol Fe^2+^/100 g FW, of chestnut and commercial feed used in the finishing pig diet.

The antioxidant capacity obtained through DPPH radical scavenging activity and FRAP assay of chestnuts’ fruit supplied in this investigation (1152.42 µg TE/g FW and 1777.49 µmol Fe^2+^/100 g, respectively) are lower than that obtained in previous studies. In this respect, Abe et al. [71] displayed a DPPH value of 1551.80 µg TE/g FW for chestnuts from Central Market of São Paulo (Brazil). For their part, Blomhoff et al. [72] and Carlsen et al. [73] obtained values ranging from 4670 to 4700 µmol antioxidant/100 g of chestnut for the FRAP assay. On the contrary, the aftermaths found in this investigation for the ABTS test (9379.74 µg TE/g FW) were much higher than those reported in other works, in which values between 136.66 and 778.4 µg TE/g FW were shown [44,74].

With respect to commercial feed, the values found for DPPH, ABTS, and FRAP assays were 957.33 µg TE/g FW, 7613.44 µg TE/g FW, and 1946.09 µmol Fe^2+^/100 g FW, respectively. There is little information in literature regarding the antioxidant capacity of feed for pigs. However, a previous study carried out by Smet et al. [75] found values between 12,500 and 68,800 µmol Fe^2+^/100 g for the FRAP test in a grain-based feed supplemented with α-tocopherol acetate, which were much higher than those shown by the commercial feed supplied in our research. Moreover, the antioxidant activities displayed for commercial feed were lower than those found in the main cereals that make up the elaborated nourishment (barley and maize). For example, in the case of DPHH radical scavenging, values between 1419.14 and 16,400.10 µg TE/g for barley [62,76] and quantities of 12,314 µg TE/g for maize [77] were observed. In addition, the ABTS test showed higher values for barley feed (8174.1–12,917.8 µg TE/g) than for the analyzed feed in this research [76]. Contrarily, the commercial feed showed a higher ABTS value than in the case of corn, where values between 1146.3 and 1551.4 µg TE/g were found [78].

As can be seen in Table 2, there are only significant (*P* < 0.001) differences between the diets in the case of the ABTS radical scavenging activity analysis, where the chestnut obtained a higher antioxidant activity than the feed (9379.74 vs. 7613.44 µg TE/g FW). Moreover, the DPPH values were also higher for the fruit than for the commercial feed (1152.42 and 957.33 µg TE/g FW, respectively), although in this case, the difference was not significant (*P* > 0.05). These events contrast with the values obtained for TPC and total flavonoids for the different feedings (Figure 1 and Figure 2), since both groups of compounds were significantly (*P* < 0.001) higher in commercial feed. However, it is known that the antioxidant power does not depend only on the concentration of antioxidant substances, but also depends on many structural elements such as the number and location of hydroxyl groups linked to the aromatic ring, and the nature and position of the substituent patterns [79]. In this way, it could be affirmed that the polyphenols and flavonoids present in the chestnut, although they are in a lower concentration, have a greater antioxidant efficiency than their counterparts in commercial feed.

Additionally, in contrast to the results obtained for DPPH and ABTS assays, the outcomes found for FRAP method in chestnut fruit were lower than the activities observed in commercial feed (1777.49 and 1946.09 µmol Fe^2+^/100 g FW, respectively), even though these differences were not significant (*P* > 0.05). This occurrence could suggest the existence of thiol groups in chestnuts, since one disadvantage of FRAP method is the fact that it does not react with thiols [72], unlike the other antioxidant methods used (DPPH and ABTS).

### 3.5. Correlation Analysis in Feed Used in the Finishing Diet of Celta Pigs

In order to clarify the relationship between the TPC and total flavonoids and the antioxidant activities in the diets supplied, a correlation analysis was conducted, and the results were shown in Table 3. In chestnuts, a positive and significant correlation between TPC and total flavonoids was observed (r = 0.903; *P* < 0.01), which is consistent with the outcomes reported by Živković et al. [80] for different parts of chestnuts. Additionally, in our study, these two groups of compounds correlated significantly with DPPH radical scavenging activity (r = 0.794; *P* < 0.05 for TPC; and r = 0.895; *P* < 0.01; for total flavonoids) and with FRAP values (r = 0.974; *P* < 0.01 for TPC; and r = 0.973; *P* < 0.01 for total flavonoids) in the case of chestnuts. Similar aftermaths were obtained by Abe et al. [71] and Dudonné et al. [81], since they observed a high correlation between TPC and antioxidant capacity of different nuts and plants. In this way, it is confirmed that phenols and flavonoids are closely correlated with the antioxidant activity of chestnuts, being the major contributors to the antioxidant properties of this fruit. However, the fact that correlation of TPC with FRAP was higher than with the DPPH could again suggest the additional existence of thiol groups that exert antioxidant activity in chestnut, apart from phenols, which are taken into account in the DPPH assay. In spite of these differences, the DPPH radical scavenging activity and FRAP method showed an acceptable correlation (r = 0.847; *P* = 0.01), which indicates that these two assays may be comparable techniques in the evaluation of the antioxidant capacity of chestnuts. On the contrary, the results obtained for ABTS radical scavenging activity are negative and significantly correlated with TPC (r = −0.838; *P* < 0.01) and total flavonoids (r = −0.834; *P* < 0.01) in chestnut feed. These outcomes are inconsistent with the report by Dinis et al. [60], where a positive correlation between both TPC and total flavonoids and the antioxidant activities determined with ABTS was found. Additionally, ABTS values are negative and significantly correlated with DPPH (r = −0.832; *P* < 0.01) and FRAP (r = −0.861; *P* < 0.01) methods. These findings are surprising, since they are in disagreement with those obtained in previous studies, where ABTS values were always positively related to the DPPH and FRAP methods [81,82,83].

On the other hand, in commercial feed, the TPC and total flavonoids are not correlated (r = 0.092; *P* > 0.05), which demonstrated that the amount of total flavonoids is not significant in the concentrated diet despite their concentration in this nourishment (Figure 2). Furthermore, these total flavonoids are not responsible for the antioxidant capacity of the commercial feed, since low correlations have been observed with the values obtained for DPPH (r = −0.066; *P* > 0.05), ABTS (r = 0.061; *P* > 0.05) and FRAP (r = −0.413; *P* > 0.05). Hence, although the commercial feed has a higher concentration of total flavonoids than chestnuts (32.18 vs. 8.58 mg CAE/100 g FW), these compounds are less important than in the natural kernel as they do not show antioxidant activity. Thus, the importance of the structure and integrity of antioxidant compounds is once again highlighted [79]. Regarding TPC, this value has an excellent positive correlation with the ABTS (r = 0.960; *P* < 0.01) for commercial feed, a fact that differs widely from what happened in chestnuts and that reveals the presence of very different antioxidant compounds in both diets. Additionally, oppositely to ABTS radical scavenging activity, TPC does not have a good correlation with the antioxidant capacity determined by DPPH (r = 0.298; *P* > 0.05) and FRAP (r = 0.358; *P* > 0.05) for commercial feed. These low correlations show that TPC does not represent the principal basis for the antioxidant capacity of the compound feed, unlike chestnuts. Therefore, it is expected that the commercial feed contains other types of substances, different than phenols, which are responsible for its antioxidant capacity. Since the commercial feed is artificially supplemented with vitamins, provitamins, and chemically defined substances analogous to Vitamin A, D_3_, and E (6500, 1500, and 15 IU/kg for Vitamin A, D_3_, and E, respectively), some of the artificially added compounds could contribute to the antioxidant activity of this nourishment [68,84,85]. Finally, the correlation between the three methods used to determine the antioxidant capacity has proven not to be very good in any of the cases for commercial feed (0.379 ≥ r ≤ 0.483; *P* > 0.05), which indicates that these techniques are not comparable to each other in the analysis of the antioxidant capacity of commercial feed.

### 3.6. Antioxidant Activity of Different Celta Pig Locations

DPPH and ABTS radicals scavenging activity and FRAP assay were used to screen the antioxidant properties of different pig muscles (*Longissimus thoracis* et *lumborum*, *Psoas major*, and *Biceps femoris*) and liver, in the same way as in the chestnuts and commercial feed diets. Table 4 displayed the effect of diet and location on DDPH and ABTS radical scavenging activity and FRAP values of these pig locations. To our knowledge, limited data about the antioxidant activity in pig meat were reported in literature, being difficult to make comparisons.

Results presented from this research reveal a significant (*P* < 0.05) effect of diet supplied on the antioxidant activity in all pig locations, except for the antioxidant capacity determined by ABTS in the liver. Specifically, the locations from pigs fed commercial feed showed a significantly higher antioxidant activity than that obtained for locations from pigs fed chestnut. These outcomes are in accordance with the muscle and liver contents of phenolic compounds previously reported in both diets (Figure 3), since these substances were found in higher concentration in pigs fed commercial feed. At the same time, these findings are consistent with those obtained for muscles of Iberian pigs, where it was observed that pigs supplemented with feeds that had a higher content of antioxidant compounds showed greater antioxidant capacities [48,61]. In addition, these aftermaths agree with those obtained in a previous study of our research group, where it was observed that the inclusion of chestnut in the finishing diet of Celta pigs increased lipid oxidation of *Biceps femoris* muscle cooked through different culinary techniques compared with the muscles of pigs fed with commercial feed [86]. This fact could be justified with the findings of the present investigation, meaning it is seen that tissues of pigs fed commercial feed has a higher TPC and higher antioxidant activities. However, despite this agreement, the greater antioxidant capacity found in the locations of pigs fed with commercial feed is in disagreement with the results obtained for the antioxidant activity of both diets, since it was observed that chestnuts had a greater antioxidant capacity than feed when analyzed by the DPPH and ABTS test (Table 2). This incident could be due to the limited absorption that some of the antioxidant compounds present in the chestnut could suffer, since, for example, it has been observed that substances that have thiol groups, which show antioxidant capacity, are absorbed in a limited way [87]. Furthermore, the higher antioxidant activity found in pigs fed commercial feed also contrasts with the results obtained in several investigations on antioxidant status of pigs fed chestnut, because several authors showed that the inclusion of chestnut in the finishing diet improved lipid oxidation in different pig matrices [24,26,27,28]. Simultaneously, our investigation also does not agree with other works that did not show any significant difference in lipid oxidation after using chestnuts in pig fattening [30,88,89].

In relation to the different location, this parameter significantly (*P* < 0.001) affected the antioxidant activities of meat and liver, regardless of the method used. In spite of that, the antioxidant capacities obtained for the muscles analyzed (*Longissimus thoracis* et *lumborum*, *Psoas major*, and *Biceps femoris*) were very similar to each other, with the greatest difference being found between the muscles and the liver. In this sense, the liver showed antioxidant capacity values much higher than those observed in the muscle tissue in both feedings, which could be due to the liver having interesting amounts of compounds such as vitamins [66,67]. Specifically, for the DPPH analysis, the values showed for the muscles were between 85.77–97.55 µg TE/g FW for the pigs fed chestnut and between 124.23–131.78 µg TE/g FW for pigs fed commercial feed, with the lowest value corresponding to *Longissimus thoracis* et *lumborum* muscle in both diets. Meanwhile, the highest DPPH value was for liver (669.08 and 881.64 µg TE/g FW for pigs fed chestnut and commercial feed, respectively). In an identical way, the antioxidant capacity values obtained by the FRAP method showed very similar ranges between the muscles (between 41.58 and 53.19 µmol Fe^2+^/100 g FW for pigs fed chestnut, and between 54.96 and 63.06 µmol Fe^2+^/100 g FW for pigs fed commercial feed), again, the *Longissimus thoracis* et *lumborum* muscle being the one with the least antioxidant activity of the three muscle tissues. Using the FRAP method, it was also the liver that had the highest antioxidant activity (659.46 and 692.52 µg TE/g FW for chestnut and commercial feed liver, respectively). On the other hand, in the case of the ABTS analysis, the values found for the muscle tissues showed greater differences between them. Thus, levels of 343.76, 503.94, and 625.83 µg TE/g FW for *Biceps femoris*, *Longissimus thoracis* et *lumborum*, and *Psoas major* from chestnut pigs were obtained, respectively; and amounts of 395.15, 831.21, and 932.10 µg TE/g FW for *Biceps femoris*, *Psoas major*, and *Longissimus thoracis* et *lumborum* from commercial feed pigs were displayed, respectively. In addition, the highest ABTS values were again for the liver (1889.67 and 2047.10 µg TE/g FW for chestnut and commercial feed liver, respectively).

### 3.7. Correlation Analysis of Different Celta Pig Locations

The Pearson’s coefficients between the TPC and the antioxidant activities analyzed by DPPH, ABTS, and FRAP assay of different Celta pig locations were presented in Table 5. The antioxidant activities measured with the three methods were positively correlated with TPC, except for DPPH, ABTS, and FRAP in *Biceps femoris* muscle from pigs fed commercial feed and DPPH and FRAP in liver from both diets. However, in general, these correlations are not very high, which suggests that there are other substances with antioxidant activity distinct from phenolic compounds in muscle tissues and liver of pigs.

This fact agrees with the premise that the presence of phenols in animal tissues is poor and is relegated to the consumption of vegetal products and their subsequent accumulation [33]. Additionally, the liver stands out for this low correlation in both diets, which may be due to the high presence of other compounds such as vitamins, which also have antioxidant activity [66,67]. Notwithstanding, it should be noted that among the three methods used, ABTS is the one that generally presents a greater correlation with the TPC, being significant (*P* < 0.01) in the case of *Longissimus thoracis* et *lumborum* and *Psoas major* muscle from pigs fed both diets and in the case of liver in pigs fed chestnut.

On the other hand, the correlation between the methods is not very high either, which suggests the existence of very different compounds that react differently to the distinct reactants used in the different techniques. This highlights the difficulty in comparing different methods for assessing antioxidant activity in animal tissues. Nevertheless, making a general analysis for all the locations, the existing correlation between the DPPH and the FRAP is the most suitable for making comparisons.

## 4. Conclusions

This study has determined that commercial feed provides a higher total phenol content and total flavonoids to the Celta pig’s diet when compared to chestnut. However, this fact has not been reflected in the antioxidant capacity assessed in commercial feed, since it has been found that the chestnut had a higher antioxidant capacity when measured by the DPPH and ABTS radical scavenging activity, although only significant differences were displayed through ABTS assay. Furthermore, it has been shown that phenolic compounds, including flavonoids, are the most responsible for the antioxidant capacity of chestnuts. Meanwhile the presence in commercial feed of other chemical compounds, which also exert antioxidant activity, has been revealed. Moreover, the aftermaths of antioxidant capacity obtained for chestnut showed the possible presence of compounds with thiol groups that could display antioxidant activity in this fruit. On the other hand, this research has observed that commercial feed significantly increases the total phenol content, as well as improves the antioxidant activity of different muscles locations (*Longissimus thoracis* et *lumborum*, *Psoas major*, and *Biceps femoris*) and liver of the Celta pig breed. Simultaneously, it has been demonstrated that in pig tissues, there are also different compounds other than phenols, which have antioxidant capacity.

In addition, it has been found that the different methods for determining the antioxidant capacity in chestnut showed a better correlation between them than the commercial feed, which could make comparisons between these two diets difficult. At the same time, the existing correlations between DPPH, ABTS, and FRAP in meat were also shown to be low in muscle tissue and liver, highlighting the difficulty of comparing techniques in these animal tissues.

## Figures and Tables

**Table 1:** Composition of the commercial feed used in the feeding of pigs.

Component	(%)	Supplements	UI (Vitamins) or mg (Minerasls)/kg
Crude protein	16.01	Vitamin A	6500
Crude fiber	5.01	Vitamin D3	1500
Crude fat	2.98	Vitamin E	15
Crude ashes	5.83	Manganese sulphate	40
Lys	0.72	Zinc oxide	60
Met	0.25	Iron sulphate	50
Thr	0.12	Copper sulphate	15
Trp	0.01	Potassium iodide	0.5
Ca	0.88	Sodium selenite	0.1
P	0.61		
Na	0.17		

**Table 2:** Antioxidant activity of chestnut and commercial feed used in the finishing diet of Celta pigs (mean ±standard error of three determinations in each feed).

	Chestnut	Commercial Feed	SEM	F
**Antioxidant Activity**				
**DPPH** (µg TE/g FW)	1152.42 ± 89.84	957.33 ± 101.50	69.878	ns
**ABTS** (µg TE/g FW	9379.74 ± 183.61	7613.44 ± 139.96	241.704	***
**FRAP** (µmol Fe^2+^/100 g FW)	1777.49 ± 124.72	1946.09 ± 24.45	64.953	ns

FW: fresh weight. TE: Trolox equivalents. SEM: standard error of the mean. F: significance; *** (*P* < 0.001); ns: no significance.

**Table 3:** Pearson correlation coefficients between antioxidant activity, total flavonoids, and total phenol content of chestnuts and commercial feed used in the finishing diet of Celta pigs.

	ABTS	FRAP	TPC	TF
**Chestnut**				
DPPH	−0.832 **	0.847 **	0.794 *	0.895 **
ABTS		−0.861 **	−0.838 **	−0.834 **
FRAP			0.974 **	0.973 **
TPC				0.903 **
**Commercial Feed**				
DPPH	0.379	0.483	0.298	−0.066
ABTS		0.432	0.960 **	0.061
FRAP			0.358	−0.413
TPC				0.092

TF: total flavonoids. TPC: total phenol content. * (*P* < 0.05); ** (*P* < 0.01).

**Table 4:** Effect of the inclusion of chestnut in the finishing diet and of location on antioxidant activity of different pig carcass tissues (mean ± standard error of nine carcasses in each feeding group).

Location	*Longissimus thoracis* et *Lumborum*	*Psoas major*	*Biceps femoris*	Liver	SEM	L	LxF
**Antioxidant Activity**							
**DPPH** (µg TE/g FW)							
Chestnut pigs	85.77 ± 3.27 ^a^	97.55 ± 2.38 ^a^	97.42 ± 2.02 ^a^	669.08 ± 18.03 ^b^	29.927	***	***
Commercial feed pigs	124.23 ± 2.78 ^a^	125.76 ± 2.55 ^a^	131.78 ± 2.16 ^a^	811.64 ± 12.91 ^b^	35.329	***	
SEM	3.879	2.939	3.248	16.265			
F	***	***	***	***			
**ABTS** (µg TE/g FW)							
Chestnut pigs	503.94 ± 19.90 ^b^	625.83 ± 19.35 ^c^	343.76 ± 10.37 ^a^	1889.67 ± 58.32 ^d^	74.579	***	***
Commercial feed pigs	932.10 ± 30.71 ^b^	831.21 ± 45.42 ^b^	395.15 ± 14.27 ^a^	2047.10 ± 67.58 ^c^	75.456	***	
SEM	40.430	29.889	9.717	45.960			
F	***	***	**	ns			
**FRAP** (µmol Fe^2+^/100 g FW)							
Chestnut pigs	41.58 ± 1.40 ^a^	53.19 ± 0.87 ^a^	52.64 ± 1.32 ^a^	659.46 ± 7.66 ^b^	31.429	***	ns
Commercial feed pigs	54.96 ± 1.26 ^a^	63.06 ± 0.98 ^a^	59.09 ± 0.78 ^a^	692.52 ± 13.00 ^b^	32.714	***	
SEM	1.464	1.055	0.932	7.942			
F	***	***	***	*			

FW: fresh weight. TE: Trolox equivalents. SEM: standard error of the mean. F: significantly different values as influenced by feeding: * (*P* < 0.05); ** (*P* < 0.01); *** (*P* < 0.001); ns: no significant difference. L: significantly different values as influenced by location. ^a–d^ Means within the same row not followed by the same letter differ significantly (*P* < 0.05) (influence of location).

**Table 5:** Pearson correlation coefficients between total phenol content and antioxidant activity of different locations of Celta pigs fed chestnut and commercial feed.

	ABTS	FRAP	TPC
**Chestnut Pigs**			
*Longissimus thoracis et lumborum*			
DPPH	0.488 *	0.401	0.222
ABTS		0.304	0.621 **
FRAP			0.549 *
*Psoas major*			
DPPH	0.243	0.389	0.143
ABTS		−0.020	0.637 **
FRAP			0.393
*Biceps femoris*			
DPPH	0.545 *	0.447	0.527 *
ABTS		0.045	0.413
FRAP			0.303
Liver			
DPPH	−0.171	0.535 *	−0.336
ABTS		−0.470 *	0.628 **
FRAP			−0.351
**Commercial Feed Pigs**			
*Longissimus thoracis et lumborum*			
DPPH	−0.045	0.164	0.008
ABTS		0.526 *	0.783 **
FRAP			0.688 **
*Psoas major*			
DPPH	0.354	0.495 *	0.444
ABTS		0.711 **	0.918 **
FRAP			0.633 **
*Biceps femoris*			
DPPH	0.224	0.386	−0.426
ABTS		−0.033	−0.257
FRAP			−0.232
Liver			
DPPH	−0.089	0.395	−0.003
ABTS		−0.387	0.433
FRAP			−0.153

TPC: total phenol content. * (*P* < 0.05); ** (*P* < 0.01).

## Data Availability

Not applicable.
